# Body composition in males with adolescent idiopathic scoliosis: a case–control study with dual-energy X-ray absorptiometry

**DOI:** 10.1186/s12891-016-0968-0

**Published:** 2016-02-29

**Authors:** Weijun Wang, Zhiwei Wang, Zezhang Zhu, Feng Zhu, Yong Qiu

**Affiliations:** Spine Surgery, The Affiliated Drum Tower Hospital of Nanjing University Medical School, Zhongshan Road 321, Nanjing, 210008 China

**Keywords:** Idiopathic scoliosis, Body composition, Lean mass, Bone mineral content, Body weight

## Abstract

**Background:**

In contrast to the well-characterized body growth and development of females with adolescent idiopathic scoliosis (AIS), the pubertal growth pattern of male patients has not been well-documented. Recently, significantly lower body weight (BW) and body mass index (BMI) were reported in males with AIS, and were thought to be related to curve progression. A case–control study was carried out to characterize the body composition and bone status of males with AIS, with the aim of gaining a better understanding of lower BW among these patients.

**Methods:**

Forty-seven males with AIS and forty age- and gender-matched healthy controls were recruited. Standing height (SH) and BW were measured. The SH of the males who had AIS was corrected using Bjure’s equation, and then the BMI was calculated. Body composition, including subcranial fat mass (FM), lean mass (LM), and bone mineral content (BMC), and bone mineral density (BMD) were analyzed using dual-energy X-ray absorptiometry. The LM index (LMi) and the FM index (FMi) were calculated by dividing the FM and LM by the square of the SH. Logistic regression analysis was employed for comparison between AIS and controls.

**Results:**

The AIS patients had comparable age and Tanner staging for pubic hair as the controls. After adjustment for age, the AIS patients showed comparable SH but significantly lower BW and BMI than that of the controls. The LM, LMi, BMC and BMD were also significantly lower in the AIS patients than in the controls. However, the difference in BMC between two groups was not significant by adjusting for age, FM and LM.

**Conclusion:**

The male AIS patients showed abnormal body composition, presenting as significantly lower LM than the controls. The lower BMC observed in the patients might due to the abnormal body composition.

## Background

Adolescent idiopathic scoliosis (AIS) is the most common spinal deformity occurred during puberty, with predominant curve progression in females [1]. The anthropometric phenotypes of female AIS patients have been well-documented in studies. These patients are taller, have a longer arm spam, and have a lower body weight (BW) and body mass index (BMI) [2–4]. In addition, female AIS patients have been found to have abnormal bone growth and metabolism, and the related pathogenesis has been widely studied, although the etiology is still unclear [1, 5, 6]. In contrast, the anthropometric characteristic of male AIS patients have been only recently reported in two studies [7, 8]. The male AIS patients were found to have significantly lower BW and BMI during and after the peak of pubertal growth than age- and gender-matched healthy controls, whereas their corrected standing height (SH) was comparable to [8] or higher [7] than that of the controls.

Because lower BW and BMI have been consistently found in both male and female AIS patients, examining body composition could help provide a better understanding of AIS pathogenesis. Less mesomorphism has been documented in female AIS patients by anthropometric methods [9, 10] and was found to be a risk factor for curve progression [10] Ramirez et al. [11] reported lower fat mass and fat free mass in girls with AIS using bioelectrical impedance analysis. Dual-energy X-ray absorptiometry (DXA) is the preferred method for assessing whole body bone mineral density and body composition, especially in children and adolescents with higher accuracy [12, 13] DXA could provide information for a three-compartment model of body composition that includes fat mass (FM), lean mass (LM), and bone mineral content (BMC). By assessing the body composition with DXA, Clark et al. [14] found children with reduced FM and LM at 10 years (yrs) old had higher incidence to develop scoliosis before 15 years old. However, none of previous study has focused on the body composition of male AIS patients. Hence, a case–control study was conducted using DXA aimed at understanding whether male AIS patients have abnormalities in body composition and bone status.

## Methods

### Subjects

Male AIS patients and age- and gender- matched healthy controls were recruited for this case–control study. Newly diagnosed male AIS patients who were candidates for corrective spinal surgery and had a Cobb angle in the major curve between 40° and 70° were recruited between the ages of 12 and 20 years. The exclusion criteria included the following: (1) history of treatment for scoliosis (i.e., brace, physical therapy); (2) history of metabolic disease, medical conditions or treatments that affect bone growth and metabolism; (3) history of fracture; (4) skeletal abnormality at any site except the spine; (5) physical activity >2 h per day or provisional athlete; (6) abnormal eating habits (e.g., vegetarian diet, picky eating); and (7) refusal to participate in the study. Ethical approval (No. 2015-039-01) was obtained from the Institutional Review Board of Nanjing Drum Tower Hospital before the start of the study. Written informed consent was obtained from the subjects and their parents.

As a result, 47 non-consecutive male AIS patients were recruited with an average age of 15.3 ± 2.2 years (range, 12.0–19.8 years). Among these patients, 26 had a major thoracic curve, 7 had a major thoracolumbar or lumbar curve and 14 a double major curve. The mean Cobb angle of the major curve was 52.7° ± 8.0° (range, 40°–69°). Forty healthy controls who had normal eating and physical activity habits were recruited from the local middle school. An experienced senior consultant spinal surgeon examined all of the subjects using the Adam’s forward bending test to rule out any spinal deformities. The exclusion criteria for the controls were the same as those for the AIS patients. The mean age of the controls was 15.3 ± 1.8 years (range, 12.9–19.1 years) and was comparable to that of AIS patients.

### Anthropometric measurements

Body weight and standing height were measured using standard techniques [8]. Body weight (BW) was measured to the nearest 0.1 kg using a standardized medical scale. Standing height (SH) was measured while the subjects stood upright against a wall-mounted stadiometer, with their heads positioned in the Frankfort horizontal plane and their heels against the stadiometer. SH was measured to the nearest 0.1 cm. The body mass index (BMI) of the controls was calculated as the weight (kg) divided by the height (m) squared (kg/m^2^). For the AIS patients, corrected SH was computed by adjusting the trunk loss using Bjure’s equation (log y = 0.011x - 0.177, where y is the reduction in trunk height (cm) caused by the spinal deformity and x is the Cobb angle of the primary curve) [15]. Corrected height was used to calculate the BMI of the AIS patients. Pubertal maturity was assessed by self-reported Tanner staging for pubic hair distribution.

### Body composition assessment

A well-trained technologist performed DXA scans in an anteroposterior position using a Lunar iDXA bone densitometer (GE Lunar, Madison, WI, USA) that was equipped with iDXA version 10.40 software. A whole body scan was performed on each AIS patient and control. The DXA scan provided the following measurements: subcranial (total body less head, TBLH) FM, LM, BMC and bone mineral density (BMD) of the whole body. Because SH has been shown to be a significant confounder of body composition, the normalization of body composition factors according to body height is recommended [16]. Hence, the FM index (FMi) and the LM index (LMi) were calculated by dividing the FM and LM by the square of the SH in the control and the corrected SH in the AIS patients. The control spine phantom scan was performed daily with a long-term (>7 y) coefficient of variance between 0.33 % and 0.40 %. According to the manufacturer’s technical data, each scan requires approximately 13 min and has an effective radiation dose of 3 μSv [17].

### Statistical analysis

Data analysis was performed using SPSS for windows (Version 13.0; SPSS Inc., Chicago, IL, USA). Logistic regression was used to compare the differences in the measured data between AIS and control. In addition, the adjusted odds ratios (ORs) and 95 % confidence intervals (CIs) were calculated by controlling confounding factors to identify the abnormalities in body composition and bone status in male AIS patients. Minimally adjusted ORs were adjusted for age, while fully adjusted ORs were additionally adjusted for SH, FM and LM. The level of significance was defined as *p* ≤ 0.05.

## Results

The AIS patients and controls showed comparable self-reported Tanner stage for pubic hair (3.9 ± 0.6 vs 4.0 ± 0.7, *p* = 0.953). The anthropometric and body composition data of the patients and controls were summarized in Table 1. By adjusting for age, no significant difference was revealed between AIS and controls in SH. However, patients with AIS showed significantly lower BW and BMI than those of the controls.

**Table 1:** Logistic regression analysis on the measurements between male AIS patients and the controls

	Control	AIS	*p* for difference	Minimally adjusted OR (95 % CI), *p*	Fully adjusted OR (95 % CI), *p*
SH (cm)	171.6 ± 8	168.6 ± 9.9	0.131	0.963 (0.810–1.146), *P* = 0.674	
BW (Kg)	63.1 ± 15.8	52.6 ± 9	**<0.001**	**0.517 (0.326**–**0.819),** ***P*** **= 0.005**	
BMI (Kg/m^2^)^ǂ^	21.3 ± 4.3	18.5 ± 2.6	**<0.001**	**0.621 (0.428**–**0.902),** ***P*** **= 0.012**	
Fat Mass (Kg)	12.8 ± 9.3	9.6 ± 5.3	0.049	0.671 (0.433–1.040), *P* = 0.074	1.652 (0.796–3.428), *P* = 0.178
Lean Mass (Kg)	43.7 ± 7.6	36.2 ± 6.8	**<0.001**	**0.420 (0.259**–**0.682),** ***P*** **< 0.001**	**0.117 (0.041**–**0.333),** ***P*** **< 0.001**
BMC (Kg/cm)	1.8 ± 0.3	1.5 ± 0.3	**<0.001**	**0.397 (0.241**–**0.656),** ***P*** **< 0.001**	0.373 (0.097–1.438), *P* = 0.152
BMD (g/cm^2^)	0.93 ± 0.142	0.819 ± 0.069	**<0.001**	**0.455 (0.288**–**0.719),** ***P*** **= 0.001**	**0.272 (0.093**–**0.801),** ***P*** **= 0.018**
FMi (Kg/m^2^)	4.29 ± 2.92	3.37 ± 1.82	0.078	0.868 (0.729–1.033), *P* = 0.111	
LMi (Kg/m^2^)	14.78 ± 1.99	12.66 ± 1.94	**<0.001**	**0.736 (0.610**–**0.888),** ***P*** **= 0.001**	

*SH* standing height, *BW* body weight, *BMI* body mass index, *BMC* bone mineral content, *BMD* bone mineral density, *FMi* fat mass index, *LMi* lean mass index, *OR* odds ratio, *CI* confidence interval

^ǂ^ The SH of AIS patients has been corrected by Bjure’s equation, and all the other analysis was carried out by the corrected height instead of measured height

ORs are per SD increase in height, weight, or body composition. Minimally adjusted ORs are adjusted for age. Fully adjusted ORs are additionally adjusted for standing height, fat mass and lean mass. Staticial significances were  highlighted in bold

(In logistic regression, control is set as “0” and AIS is “1”)

For the body composition, the AIS patients had significantly lower LM, LMi and BMC than the controls by adjusting for age. In contrast, the FM and FMi were comparable between two groups. By further adjustment for SH, FM and LM, the BMC become comparable between two groups. In contrast, the BMD in patients with AIS was significantly lower than that in the controls, either adjusted for age only, or together with SH, LM and FM.

## Discussion

The anthropometry and body composition of 47 male AIS patients were studied and compared to those of 40 age- and gender- matched healthy controls. The patients showed significant lower BW and BMI values. After adjusting for height loss using Bjure’s equation, the standing height of the AIS patients was comparable to that of the controls. The AIS patients had significantly lower subcranial LM, LMi and BMC than the healthy controls. These findings suggested that male AIS patients have abnormal body composition. In addition, the comparable BMC but significantly lower subcranial BMD observed in the males with AIS than those of healthy controls by adjustment for SH, FM and LM indicated that they may be abnormal bone metabolism in these patients.

Curve progression in AIS patients mainly occurs during puberty and is highly correlated with the velocity of longitudinal growth [2, 4, 18–20]. Abnormal pubertal growth has been documented in both female and male AIS patients. Female AIS patients showed significantly greater corrected standing height and longer arm spans after the onset of puberty than their age- and gender-matched healthy peers [4]. In addition, they showed significantly lower BW and BMI and a higher incidence of underweight than healthy control [21]. In contrast, only two studies have focused on the anthropometry of males who have AIS [7, 8]. Our previous cross-sectional study, which included 332 male AIS patients and 356 controls between the ages of 11 and 19 years, found that the AIS patients had comparable SH and arm span to the controls at each age group [8]. Another study, conducted by Oh et al. [7], compared 420 male AIS patients, who were 19 years of age, with 409 age-matched controls, showed greater SH in the AIS patients than that of the controls (176.1 cm vs. 174.7 cm) although the difference was small. Our previous study [8] also showed that compared to the controls, the male AIS patients who were older than 14 years had significantly lower BW and that the patients between the ages of 15 and 17 years had significantly lower BMI. In contrast, Oh et al. [7] found that the male AIS patients had significantly lower BW and BMI than the controls. In the present study, the subjects in both groups were newly recruited, and the results are consistent with previous findings; the male AIS patients showed comparable corrected SH to that of the controls, whereas their BW and BMI were significantly lower than those of the controls.

Since lower BW and BMI have been commonly observed in patients with AIS, we thought it would be interesting to examine their body composition. The morphologic somatotypes of the human body can be classified as endomorphic, mesomorphic and ectomorphic [22]. By analyzing the morphologic somatotypes through the measurement of skinfold thickness, LeBlanc et al. [10] found that the girls with progressive AIS were less mesomorphic than the age- and gender-matched controls. Mesomorphism results from the development of bone, cartilage, ligament and muscle; hence, the finding by LeBlanc et al. [10] suggested that bone and muscle mass are less developed in patients with progressive AIS. By comparing the body composition profiles of 52 female AIS patients to those of 92 age-matched healthy female controls, Barrios et al. [9] also found that the female AIS patients had a lower mesomorphic component. Moreover, their results showed a significantly higher ectomorphic component and a marginally lower endomorphic component in the AIS patients than in the controls. Because lower endomorphism is related to delayed maturation and higher ectomorphism is related to delayed onset of puberty, the observed somatotypes in girls with AIS might be related to their delay in puberty onset and physical maturation in terms of skeletal development [9, 23]. In addition to analyzing the somatotypes of AIS patients, Ramirez et al. [11] used bioelectrical impedance analysis to examine the body composition of 27 female AIS patients who were candidates for corrective spinal surgery. They compared their results with data from a study of 488 age- and gender-matched healthy controls and found that the AIS patients had significantly lower BW, BMI, FM and FFM (fat free mass, same as LM in the present study). In addition, the AIS patients showed significantly lower FMi and FFMi than 25 locally recruited healthy girls. The application of DXA on body composition assessment has been found more accurately than other methods, such as skinfold thickness measurements and impedance analysis [12]. Recently significant association between altered body composition assessed by DXA and the occurrence of scoliosis during puberty was reported by Clark et al. [14] with a population-based prospective longitudinal study. They found that the lower FM and LM at 10 years suggested higher incidence of scoliosis before 15 years, and per standard deviation increase in FM at 10 years would result in 14 % reduced risk of scoliosis at 15 years by adjusting for age and gender, while per standard deviation increase in LM could result in 20 % reduced risk of scoliosis at 15 years by adjusting for age, gender, FM and leg length. In the study by Clarks et al. [14], the etiology of scoliosis occurred during puberty was not clarified although most of them should be idiopathic, and the severity of scoliosis was unknown. With the great interest in body composition of male with AIS, whole body scans by DXA were conducted. After adjustment for age, we observed significantly lower BW, BMI, LM and LMi in the male AIS patients than in the controls. These findings were in agreeing to those reported in female AIS patients [10, 11], although lower FM was not retrieved. Our results support the hypothesis that male AIS patients have an altered body composition compared to that of healthy controls. Moreover, the abnormalities in body composition that have been consistently observed in male and female AIS patients support that lower BW, BMI and LM were a general phenotype of AIS. In addition, this phenotype might be existed before the occurrence of scoliosis during puberty [14].

Significantly lower BMC and BMD were found in the male AIS patients than that of the controls by adjusting for age in the present study. The bone mineral status of female AIS patients has been widely studied, and the most comprehensive data have been reported by a research group in Hong Kong [24–31]. In their studies, female AIS patients showed a significantly lower percentage (28–33 %) of bone mass and this was found to be a systemic phenomenon that could persist into skeletal maturity [24–27]. They also found that the patients suffered from diminished bone quality [28]. Moreover, osteopenia, which was detected by DXA and the bone stiffness index, which was assessed by quantitative ultrasound, were found to be prognostic factors of curve progression in females with AIS [29, 30]. In contrast, none of the previous studies have focused on the bone status of male AIS patients [31]. The present study showed that the male AIS patients had significantly lower BMC and BMD when compared with age- and gender- matched healthy male controls. However, due to the small size in the control group, the reference Z-score could not be calculated. Hence, the incidence of osteopenia and osteoporosis in the male AIS patients could not be determined in the present study. Weight-bearing is highly associated with bone mineral accrual during adolescence due to the additional mechanical loading during physical exercise and the resulting muscle-mediated effects [32]. In children, total and areal BMD values have been reported to be highly related to body mass, FM, FFM and BMI [33]. In studying the bone mineral status of AIS patients, Szalay et al. [34] suggested that the decreased BMD in scoliosis patients might due to lower BMI and that patients with normal or heavy weight were much less likely to have lower BMD than controls. In the present study, the BMC in patients with AIS was no longer different from that of controls by adjusting for the age, SH, FM and LM. This finding was in agree with previous studies and supported that lower weight might play an important role in the lower bone mineral content of male AIS patients. Several studies have demonstrated that LM was more important than FM in contributing to bone mineral accumulation [13, 35, 36] and that the contribution of FM was not consistent [37]. Our results also showed significantly lower LM in male AIS patients than the controls when adjusted for age and FM. After when further adjusted for age, SH, LM and FM, the difference in BMD between male AIS patients and controls was still significant, suggesting there may be abnormal bone metabolism in the male AIS. Thus, it could be interest to carry out further study focusing on the bone metabolism in male AIS.

Several limitations cannot be neglected in the present study. Both body weight and bone mineral status could be affected by several factors, such as nutritional status, eating habits, physical activity level and weight-bearing. These confounders were neither quantified nor controlled for in our study. In addition, the application of Adams forward bending test to rule out spinal deformity may have bias since small spinal curvature could be missed without X-ray films examination, although this test was done by experienced senior consultant spine surgeon. Moreover, this study had a relatively small sample size when compared with the previous studies on the bone mineral status of AIS patients; this was partially due to the fact that the prevalence of AIS is rarer in males.

## Conclusions

The present study showed that male AIS patients had abnormal body composition than healthy controls. The phenotype of significantly lower BW, BMI and LM found in male AIS patients was similar to that reported in female patients. The lower BMC observed in these patients might due to the abnormal body composition. In addition to the findings by Clark et al. [14] indicated that the lower LM is presented before the onset of clinically detected scoliosis, further studies are encouraged to explore the possible etiology for the lower BW and LM in patients with AIS, and their role toward the development of scoliosis during puberty.

## Abbreviations

AIS: adolescent idiopathic scoliosis
BMC: bone mineral content
BMD: bone mineral density
BMI: body mass index
BW: body weight
DXA: Dual-energy X-ray absorptiometry
FM: fat mass
FMi: FM index
LM: lean mass
LMi: LM index
SH: Standing height
yrs: years
